# Reply to Comment on Dhiman, R. et al. Correlation of Non-Polio Acute Flaccid Paralysis Rate with Pulse Polio Frequency in India. *Int. J. Environ. Res. Public Health* 2018, *15*, 1755

**DOI:** 10.3390/ijerph16010063

**Published:** 2018-12-27

**Authors:** Rachna Dhiman, Sandeep C. Prakash, V. Sreenivas, Jacob Puliyel

**Affiliations:** 1 Department of Pediatrics, St Stephens Hospital, Delhi 110054, India; dhimanrachna48@gmail.com (R.D.); sandeepcpp25@gmail.com (S.C.P.); 2 Department of Biostatistics, All India Institute of Medical Sciences, New Delhi 10029, India; sreenivas_vishnu@yahoo.com

We thank the authors for their interest in our paper [1]. However, without providing any data or references to support their contentions, they have speculated on eight reasons why the inferences made in our publication are questionable. It appears that none of these objections are valid and we discuss each point in detail below.

1. The correspondent has drawns attention to the fact that pulse polio campaigns target children under five, whereas AFP surveillance includes all children under 15. They suggest that AFP in children aged 5 to 15 may have influenced our results wrongly.
  We disagree that it is wrong to look at AFP surveillance data under 15. First, the correspondent is mistaken in assuming that the influence of the vaccine will end abruptly at five years of age and cannot influence AFP at six years of age just because OPV is given to children under five. Furthermore, it must be remembered that OPV is a live-virus vaccine. It is known that the vaccine virus, which is excreted in sewage, can contaminate the water supply and induce both protection in unvaccinated children and can revert to a virulent form and cause vaccine-virus associated polio paralysis [2]. In this way, pulse polio in children under five could well influence AFP in children aged 5–15 years, and therefore, it is appropriate to include them in our evaluation.
  The incidence of polio in the age group 5–15 years is very low which is the rationale for limiting the vaccination to children under five years of age. In fact, the incidence of Guillain-Barré syndrome (GBS), which is ordinarily one of the main causes of non-polio AFP (NP AFP), is also much higher in children under four years. The annualized rate for GBS was 1.3/100,000 for children under four years when compared to 0.1/100,000 in the age group of 5 to 15 in the study by Winner and Evans [3]. The inclusion of the AFP rate in children 5–15 years (where the natural incidence is low) will result in the underestimation of the problem we highlighted in our paper, and the AFP rates would have been even higher if we considered only children under five.
2. The correspondent suggests that a broadened case definition and the reduced support in human resources from the WHO, with fewer visits by these officers to the reporting centers, may correlate to non-polio AFP rates in recent years.
  The correspondent has provided no data to substantiate this assertion. The broadening of the case definition took place long before the fall in AFP rate seen from 2011. Moreover, a broadened definition would have resulted in an increase in the reported incidence and cannot explain why AFP rates have fallen steadily since 2011.
  The suggestion that the reporting of AFP has fallen because the number of visits by WHO experts has been scaled down is both unwarranted and mischievous. The AFP surveillance machinery in the country has been set up for meticulous reporting at great recurring cost to the Government of India. AFP cases are investigated within 48 h, which is an AFP surveillance performance indicator. All of the performance indicators from India have consistently been exemplary. It is unfortunate that the correspondents have resorted to innuendo without showing any evidence of a drop in performance from 2011 onwards.
3. The variation seen in the AFP rates in places receiving the same number of pulse polio doses was questioned.
  Area wise variations in susceptibility to vaccine preventable disease as well as the adverse effects of immunization are well known. In a study of another oral vaccine, the rotavirus vaccine, the incidence of intussusceptions was 27.7/100,000 child-years in Delhi, but was 20 times higher in Vellore, South India (581/100,000 child-years) [4].
4. It was pointed out that routine immunization coverage improved after 2013–2014, which was not taken into account in our calculations.
  We did not include routine immunization in our calculations. Routine immunization consists of three doses in the first year, one dose in the second year, and one dose in the fifth year. We considered this as a constant in all children. Having assumed full coverage from the start, we looked at the additional pulse polio doses given. As such improvements in coverage were not taken into account, they are unlikely to have significantly influenced the results.
5. It was suggested that we had not considered the global switch in 2016 from tOPV to bOPV.
  It was also stated that we had not considered the low coverage with IPV due to global shortages in our calculations.
  How this switch to bOPV that was to happen in 2016 could influence a fall in the AFP rate five years earlier in 2011, has not been explained by the correspondent.
  With regard to shortages in IPV, it must be clarified that IPV has been used very infrequently in India and so the shortages of IPV did not have any significant impact. Our calculations only considered the doses of oral vaccine given, which was not influenced by the IPV shortage.
6. The correspondent writes that the incidence of post vaccination paralysis in the literature is about one in 2–3 million doses, and that it was seen in those given the vaccine for the first time. Therefore, they say that there is no biological plausibility for the conclusion on correlation as described by us.
  This appears to be a straw man argument. We did not say that the NP AFP reported in our paper were cases of vaccine induced paralysis. Non-polio AFP, by its very definition, excludes polio vaccine induced paralysis.
7. The correspondent claims that non-polio entroviruses (NPEV) causing polio like paralysis was unaccounted for in our paper.
  This is not correct. It seems that the correspondent has not read our paper carefully. I quote from the paper:
  “We speculate that repeated doses of live vaccine virus delivered to the intestine may colonize the gut and alter the viral microbiome of the intestine, and this can result in strain shifts of enteropathogens. It is possible that new neurotropic enteroviruses colonizing the gut may induce paralysis”.
8. The correspondent suggests that in India, we do not use the classical AFP criteria practiced in Western countries, but have used NP AFP rates from the West when making our comparisons.
  We clarify that we have used the WHO recommended surveillance standards, not a ‘Western standard’. This was quoted as Reference [7] in our paper.

It would appear that the correspondent is clutching at straws to discredit our findings. We hope we have clarified all of their queries.
